# Ethnic Minority Groups’ Experiences of Suicide Bereavement: A Qualitative Exploratory Study

**DOI:** 10.3390/ijerph182211860

**Published:** 2021-11-12

**Authors:** Pauline Rivart, Verity Wainwright, Sandra Flynn, Isabelle M. Hunt, Jenny Shaw, Shirley Smith, Barry McGale, Sharon McDonnell

**Affiliations:** 1 The Centre for Mental Health and Safety, Faculty of Biology, Medicine and Health, University of Manchester, Manchester M13 9PL, UK; verity.wainwright@manchester.ac.uk (V.W.); sandra.m.flynn@manchester.ac.uk (S.F.); isabelle.m.hunt@manchester.ac.uk (I.M.H.); jennifer.j.shaw@manchester.ac.uk (J.S.); sharon.mcdonnell@suicidebereavementuk.com (S.M.); 2 Greater Manchester Mental Health NHS Foundation Trust, Manchester M25 3BL, UK; 3 Independent Advisory Panel on Deaths in Custody, London SW1H 9AJ, UK; 4 If U Care Share Foundation, Chester-le-Street, Chester DH2 2EY, UK; shirley@ifucareshare.co.uk; 5 Support After Suicide Partnership, London SE1 7NQ, UK; mcgaleb@aol.com; 6 Suicide Bereavement UK, Ramsbottom BL0 9EX, UK

**Keywords:** suicide, bereavement, ethnic minority, support, stigma, postvention

## Abstract

It is estimated that between 36,000 and 360,000 people are affected by suicide every year in the UK, and a proportion may develop depression and post-traumatic stress disorder, or engage in high-risk behaviours. Recent systematic analyses have revealed a clear gap in research on suicide bereavement in minority ethnic groups. This study aimed to understand the experiences and support needs of individuals from ethnic minority backgrounds bereaved by suicide and was the first in the UK to investigate this matter. The study was a secondary analysis of data. Participants were 7158 people residing in the UK who completed an online survey about their experiences of suicide. Free-text qualitative responses of 227 participants who did not identify as White British were analysed using thematic analysis. Four themes were identified: maladaptive coping strategies, emotional processes following suicide, lack of support from agencies, and the importance of mental health awareness. Ethnic minority groups reported a lack of support despite attempts to engage with services, noted the prevalence of stigma within ethnic minority groups, and expressed a need to tackle this. These preliminary results suggest that ethnic minority individuals require visible and accessible services that can successfully engage with and support them.

## 1. Introduction

Every year, around 6000 people die by suicide in the United Kingdom [1] and it is estimated that between six and 60 individuals are affected by each suicide [2]. However, more recent evidence suggests that up to 135 individuals could be affected [3] and it is possible that the number of people bereaved by suicide every year in the UK has been underestimated. Data from the past few years also indicate that suicide rates are increasing in the UK [1].

People bereaved by suicide experience physical and mental health issues, including a higher risk of attempting suicide and dying by suicide, developing depression and post-traumatic stress disorder (PTSD), and engaging in high-risk behaviours (such as substance abuse) [4,5]. In the UK, around 10% of individuals bereaved by suicide will attempt to take their own lives, whilst 8% will withdraw from education or resign from their job [6]. Compared to people bereaved by other sudden modes of death, higher levels of stigma, shame, and guilt are also reported in this group [7]. As a result, suicide bereavement is considered to be a highly stigmatizing type of bereavement, limiting the help-seeking behaviour of those bereaved [7,8].

Support in the UK for those bereaved by suicide, known as postvention, is limited and inconsistent [9], despite the breadth of research showing that postvention is highly valued by people bereaved by suicide and can assist in the grieving process [4,10]. Therefore, the National Institute of Care Excellence (NICE) recommended that support be made more available across the UK and that the bereaved should be fully involved in the development of postvention programs [9]. This was further acknowledged by the latest NHS Long-Term Plan which highlighted the importance of putting into place consistent support for those bereaved by suicide in the UK [11]. As part of this plan, the Department of Health and Social Care announced that funding would be provided across England to develop services offering individual or group support sessions or referrals to appropriate mental health services for the bereaved [12]. Similar strategies exist in the devolved nations of the UK [13,14,15].

Whilst postvention needs are individual, certain specific groups (e.g., children or ethnic minority groups) may have particular needs [16,17]. Understanding how and why these may differ would be beneficial to the development of postvention, for example by considering how the support needs of these individuals may be influenced by cultural beliefs (e.g., religion and spirituality [18]). Yet, there has been very little research on these groups [19]. Whilst there seems to be no significant differences between ethnic groups in terms of grieving processes [20], bereaved Black Caribbean individuals in the UK reported poorer health outcomes compared to their White counterparts, following the loss of a relative or friend to a progressive disease [21]. This outcome was associated with housing and legal issues following the loss of a loved one and suggested that individuals belonging to a minority ethnic group may have different support needs [20,21].

Additional barriers to help-seeking that are not found among White British individuals also need to be acknowledged when considering postvention interventions. Shefer et al. [22] argued that minority ethnic groups face two types of stigma when seeking help for mental health support: perceived or external stigma (i.e., perceived inequalities and discrimination within medical institutions), which can lead to feelings of mistrust towards professionals, as well as internal stigma regarding perceptions of mental health and associated feelings of shame and guilt within their community. In the UK, people from ethnic minority groups have reported that their cultural identity can be an obstacle to identifying psychological distress and seeking help for the latter [23]. As a result, general mental health provision in the UK has taken some steps towards tackling inequalities in mental health among ethnic minority groups, notably by increasing representation and diversity in mental health professionals [24]. Finally, there is evidence that ethnic minority groups are heterogeneous and differ from one another in terms of help-seeking [25] and this needs to be considered by postvention interventions.

Research has highlighted that individuals bereaved by suicide are a high-risk, vulnerable group, specifically regarding suicide ideation, but it is largely unknown how ethnic minority groups experience suicide bereavement. As a result, it is also unknown how these groups can be supported to reduce the impact of suicide bereavement on physical and mental health outcomes. This study aimed to address these gaps in knowledge by exploring the experiences of individuals bereaved by suicide in people from a minority ethnic background. Ultimately, furthering our knowledge has the potential to inform research and practice in the suicide bereavement field with regards to ethnic minorities in the UK, notably to support the development of specialized postvention services if required.

## 2. Materials and Methods

The current study presents a secondary analysis of qualitative survey data. This analysis was undertaken as part of the first author’s Master of Science degree.

### 2.1. Materials

The survey designed for the primary study included 71 questions, of which 41 were checkbox questions, 26 were free-text questions, and 4 were both checkbox and free-text questions. The questions revolved around three main topics: suicide bereavement in general and in the workplace, the impact of suicide on the bereaved, and access to support following suicide bereavement. For the current study, seven free-text questions (see Table 1) were selected for analysis by the lead researcher (first author) and reviewed by the wider research team. This selection was guided by previous literature on the impact of suicide bereavement and research on grief outcomes and help-seeking among ethnic minority groups. Questions which could potentially inform future practice and the development of postvention with these groups were also retained. The questions, the range of response length (i.e., shortest answer to longest answer), the mean response length in words, and the response frequency (i.e., how many participants responded) for each question can be found in Table 1.

### 2.2. Participants

The primary study recruited 7158 participants using convenience and snowball sampling. Participants were eligible to take part if they were aged 18 or older, lived in the UK, and perceived themselves as affected and/or bereaved by suicide. For the secondary analysis, the sample included all participants identifying as non-White British. The current study included 227 participants (3.2% of the primary sample): 174 females (77%), 48 males (21%), and 1 non-binary individual. The mean age of participants was 38.79 years old (*SD* = 12.44) and ranged from 18 to 72 years old. In terms of ethnicity, 106 (46.7%) participants identified as multiple/Mixed race, 73 (32.2%) as Asian, 32 (14.1%) as Black Caribbean or African, and 16 (7%) as belonging to any other ethnic background (including Arab and Middle Eastern). Further information on the ethnic background of participants can be found in Table 2. For nearly a fifth (18.5%) of the sample, suicide had occurred between two and five years ago, and for around a sixth (15%), it had occurred between ten and twenty years ago. The rest of the sample had been bereaved for less than six months (8.8%), between six months and a year (7.5%), between one year and two years (8.4%), between five and ten years (10.1%), and over 20 years (6.6%). One participant in six had lost a friend (17.6%), one in seven a sibling (14.1%), and one in ten (9.3%) a relative other than a parent, child, or sibling (e.g., aunt/uncle, grandparent, cousin). The rest of the sample had lost a parent (6.6%), a spouse or partner (5.3%), or a child (4.4%).

### 2.3. Procedure

The primary study advertised the survey on the Centre for Mental Health and Safety, University of Manchester, UK website and the Support After Suicide Partnership website between July 2017 and August 2018, as well as newspapers, social media, on radio and TV, conferences and by word of mouth. Participants could complete the survey online or using a paper version. The survey was preceded by the participant information sheet. The latter informed participants that completing the survey would be taken as informed consent, and that they could withdraw at any time without penalty. However, due to the anonymity of the responses, participants were informed that, once the survey was completed, it would not be possible to identify and withdraw their responses from the collected data. Information signposting individuals to support organizations was provided at the end of the survey which was designed to provide participants with support and to minimise any potential distress as much as possible. The survey was expected to take 30 min to complete.

### 2.4. Data Analysis

Thematic analysis was used to identify themes and patterns in the data. This analysis technique was chosen over other qualitative approaches as the focus of this study was on the experiences associated with suicide bereavement, rather than how participants made sense of or narrated these experiences [26]. As this was an exploratory study, the data were analyzed using an inductive approach where the experiences of the participants led to patterns and potential theory [26].

The thematic analysis was conducted using NVivo (Version 12; QSR International, 2020) and was guided by the steps described by Braun and Clarke [26]. The coding of the data was undertaken by the first author. Once all data were analyzed, codes were reviewed and similar concepts merged whilst broader ones were split into more specific codes. A codebook was created to document this process and was shared with the research team. Codes were then organized into prevalent patterns found in the sample and to answer the research question with as accurate an interpretation of the data as possible.

## 3. Results

Four themes were identified: maladaptive coping strategies, emotional processes following suicide, lack of support from agencies, and the importance of mental health awareness. Each theme will be discussed using illustrative quotes.

### 3.1. Maladaptive Coping Strategies

Participants reported engaging in maladaptive coping strategies as a consequence of suicide bereavement. This was first reflected in a number of participants recalling they had engaged in harmful behaviors, such as self-harm, suicide ideation, or suicide attempts, to try and cope with their loss. One participant reported that “in the years after her death, I have had thoughts of self-harm and thoughts of suicide although I do not think I ever would have done either of these things, the thoughts were very distressing” (participant 190). Another participant reported experiences of suicide ideation: “I have had fleeting thoughts of not wanting to go on myself” (participant 184). Experiencing harmful behaviors as a consequence of suicide bereavement was one of the most reported experiences in the sample.

Another maladaptive coping strategy adopted by suicide loss survivors was increased alcohol use. One participant explained engaging in “excessive drinking and drugs” (participant 134) and another reported that their loss had “increased my alcoholism” (participant 225). Whilst this was not as prevalent as experiences of suicide ideation or self-harm, it was one of the most reported high-risk behaviors.

These high-risk behaviors seemed at times to be related to the initial emotional reactions of participants to the death. Indeed, a majority reported anger as a significant experience associated with suicide bereavement. One participant reported feeling “awful angry at the situation” (participant 65) whilst another explained that they “felt very angry that he had done it” (participant 144). Experiences of anger in the sample seemed to be particularly intense and at times overwhelming. In some cases, this intense anger resulted in other maladaptive coping behaviors: one participant reported that their “upset and anger about the suicide as well as other problems led to dangerous driving” (participant 226). Overall, these maladaptive coping strategies seemed to be an initial response to the experienced loss, similar to acute grief.

### 3.2. Emotional Processes following Suicide

Participants reported experiencing guilt associated with their own behavior towards the deceased before the suicide, feeling that they could have done more to help or could have been more present for the deceased:

*I have a sense of guilt and shame—that is not overpowering but always lingers. I wonder what I could have done to help. I feel like I could have reached out in better ways. I knew that he was going through a tough time—and I feel like I didn’t do enough to help when I knew (participant 163).*

Several participants also reported feeling guilt regarding their inability to notice the distress the deceased had been experiencing before their death, and as a consequence felt that they had failed them: “Overriding guilt of not having known more deeply about how he was feeling or done more to help” (participant 106). As such, guilt was one of the main feelings reported by participants.

In addition, a significant number of participants reported feelings of abandonment. They felt as if they had been left behind by the deceased and were having difficulties making sense of the world without them. One participant explained that “there was no one left for me or to be proud of me because he was the only person who really parented [sic] me, so I felt hopeless in living because there was no one to make proud or happy” (participant 197). Such feelings were especially heightened in participants who reported experiencing suicide ideation before the death: “It affected me because having had my own suicidal thoughts it felt like the option had now been removed” (participant 64). This participant reported experiencing feelings of anger and resentment, as their own issues were minimized and not being attended to following the suicide. On the other hand, another participant reported that: “I think it has made me feel a bit more likely to act on suicidal feelings as I think of him having done it and not feeling in pain when I am in a lot of emotional pain” (participant 137). This participant reported having experienced severe mental health issues in the past, and their experience of losing someone to suicide seemed to have enabled them to draw parallels between their own mental health difficulties and that of their loved one. Ultimately, this resulted in them feeling increasingly vulnerable to suicide. Feelings of abandonment were reported by many participants but manifested themselves in different ways from one participant to another.

These feelings of guilt and abandonment seemed to influence the participants’ understanding of the suicide and how they made sense of it, known as ‘meaning-making’ in the literature [27]. For most participants, this was manifested by a need to replay potential scenarios. Indeed, participants reported attempts to construct a narrative to try and understand the suicide due to their feelings of guilt: “often [I] would try and replay scenarios and put myself in his shoes but found it really hard to understand how this could happen” (participant 12). Another participant who also experienced this phenomenon described it as a distressing experience: “Nothing prepares you to lose someone that way. You go insane thinking of all the times that you could have helped or done more and it consumes you” (participant 98). Arguably, the need to replay scenarios could be explained by the shock and disbelief usually associated with suicide deaths. Participants may have felt they needed to try and make sense of the decision-making of the deceased. This can also be seen as an attempt to make sense of their own perceived responsibility for the death, and to resolve issues of guilt.

The distress associated with these emotional processes suggested an obvious need for support; yet, support following suicide bereavement was found to be lacking.

### 3.3. Lack of Support from Agencies

The majority of participants reported a clear lack of support which was manifested through different scenarios. First, most participants reported a lack of awareness regarding support provision. They explained that they did not receive any support information from agencies and, as a result, had to seek support on their own. One participant reported that “if there were any support services around suicide, they were invisible to me (or very low profile)” (participant 194), suggesting that the taboo around suicide impacted the visibility and accessibility of support. This was supported by another participant who reported that “there was not much indication of what help/support was available” (participant 178). These accounts suggest that participants were proactive in attempting to access support; however, the inability to find appropriate support may have led them to experience a sense of helplessness and hopelessness, which, in turn, may have put them at an increased risk of suicide themselves.

On the other hand, although some participants reported having received some form of support information, their attempts to access support were unsuccessful. One participant reported trying to access support by calling a number of different agencies:

*There was no help for me. Charities [sic] couldn’t deal with bereavement. Other charity [sic] who are supposed to deal with bereavement just gave me phone numbers and I was constantly calling different number [sic] and it was getting me nowhere (participant 130).*

Another participant described a similar experience: “asked for help but was ignored, by many organizations, people push you from pillar to post” (participant 208). Participants reported limited empathy and compassion from agencies throughout the process of seeking help, and subsequently described having very little faith in them. This, once again, may have led to feelings of hopelessness and helplessness. Overall, the experiences reported by the participants suggested that there was no structured and coordinated approach to support provision between agencies.

Few participants accessed support and had negative experiences with professionals. Indeed, a number of these participants reported that professionals providing support for suicide bereavement had behaved in an inconsiderate manner. When asked about their experience of receiving support, one participant described it as very negative: “Dreadful—the visitor just kept saying ‘no wonder he killed himself, he had so many problems’” (participant 90). Another participant who reached out to their GP and asked to be put on medication to cope with their loss reported that “one doctor actually said to me, ‘Well, pills won’t bring your brother back will they’” (participant 203). Overall, participants explained that such experiences served to invalidate their difficulties and made them even less likely to access support.

The police were cited numerous times by participants as an agency which provided insufficient support. Participants believed that, as police officers are usually among the first responders when a suicide occurs, they should provide more support for people bereaved by suicide: “I believe the police are the first people who have contact with you when you experience suicide and they don’t give you any information where to seek support” (participant 176). Similar to other agencies, there did not seem to be a straightforward procedure following a suicide death. One participant explained that “apparently I should have had more support from the police and should have an assigned officer but [I] had nothing. I did receive a call from the police who said they were sending me a booklet which never arrived” (participant 214). This may have reinforced feelings of abandonment and isolation in participants, especially since the police are usually regarded as an agency that can provide help in times of crisis.

Overall, the lack of coordinated support made help-seeking after suicide bereavement difficult at a time when participants already felt severely distressed and, as a result, often felt unable to seek help.

### 3.4. Importance of Mental Health Awareness

A large proportion of the sample mentioned the need for mental health awareness when asked what could be improved regarding suicide bereavement support. This suggestion was the most common in the sample when participants were asked about potential areas of improvement for suicide bereavement support. This seemed to reflect the participants’ belief that there was a need for prevention before postvention. In the sample, the importance of mental health awareness was reported to serve two main purposes.

First, participants believed that increased mental health awareness could help people experiencing suicide ideation. One participant explained there is a need for “more openly available information on the importance of mental health and that there are people who have suffered the same and come through to the other side” (participant 12). There was also a belief that such awareness could also help bereaved individuals, who themselves may be at an increased risk of suicide ideation/attempt following suicide bereavement. This was reported by another participant: “there was (still is) some taboo around discussing suicide, so some education to prepare people for if/when it happens around them; an awareness issue” (participant 194). Overall, participants suggested that increased mental health awareness could help both people with mental health issues and bereaved individuals.

Second, participants suggested that mental health awareness could reduce the stigma experienced by individuals bereaved by suicide. Indeed, a number of participants reported experiencing stigma following suicide bereavement and they believed that talking more openly about mental health issues could help. One participant suggested: “have people talk and share, there is so much stigma around this from the place I come from” (participant 151, of Indian ethnicity). This was highlighted by another participant: “have to remove the stigma around the word suicide” (participant 156). More importantly, a few participants mentioned that this was even more needed among certain ethnic minority groups. Indeed, participants explained that suicide is rarely discussed in some communities and that this can reinforce stigma: “There seems to be a lack of support groups within minority ethnic groups and a lack of faith led support groups as suicide is often not talked about and the individual can feel isolated” (participant 79, of Pakistani ethnicity). This was also reported by another participant from an Asian background: “I think there should be support offered within the British Asian Community who still see it as something to hide and shameful” (participant 86, of Indian ethnicity). Participants suggested that stigma is an even more prevalent issue among people from a minority ethnic background; therefore, the need for awareness was considered more critical in this group. The words “hide”, “shameful”, and “not talked about” particularly suggested the taboo around suicide for minority ethnic groups. Several participants reported being told to get on with their lives and to not dwell on the suicide death for too long. Consequently, participants felt rejected by and isolated from their own community, which may have further impacted their ability to seek help.

## 4. Discussion

### 4.1. Findings

This study aimed to explore and understand the experiences of individuals bereaved by suicide in ethnic minority groups, specifically in relation to support needs and access to support. Four themes were identified. Participants reported a range of maladaptive coping strategies including harmful behaviors, increased alcohol use, and anger issues as a result of suicide bereavement. Participants also reported that their understanding of the suicide had been largely influenced by emotional processes following the latter which, for most participants, was guided by feelings of guilt and abandonment, leading to further distress. A lack of support was identified. Overall, participants reported that they had sought help but had received very limited support or none at all. This suggested that participants were proactive in seeking support and willing to engage with services. The need for increased mental health awareness within ethnic minority communities was also expressed, notably to tackle the stigma experienced by people bereaved by suicide, which was perceived as more significant in some communities. This finding suggested that these groups may need culture-specific support, including mental health support, to prevent issues of stigma and isolation.

The maladaptive behaviors evidenced here have been reported previously in studies looking at individuals bereaved by suicide in general. McDonnell et al. [4] reported an increased risk of engaging in suicidal behaviors, such as self-harm, and high-risk behaviors including substance abuse, unsafe driving, or sexual promiscuity. Guilt and stigma have also been found to be significant experiences of suicide bereavement, whereby individuals blame themselves for death and experience social rejection from others [8]. Previous studies in the UK have revealed that accessing support is difficult and often not immediate, and that many bereaved individuals are not offered any formal or informal support [28]. Wainwright et al. [10] have also reported that people bereaved by suicide often feel helpless and hopeless when trying to access professional help. In our sample, participants felt that professionals were not well-equipped to support them. General practitioners in the UK have previously reported that they did not feel they had adequate training to support individuals bereaved by suicide [29]. This evident need for specialized support for individuals bereaved by suicide demonstrated by existing literature was also apparent in this sample.

The experiences reported in this study seem to be in line with the literature on individuals bereaved by suicide, especially with regards to coping behaviors, poor access to support, and associated feelings of helplessness [4,10]. Whilst there may be some variation within different ethnic minority communities, the findings suggest that, overall, these groups report very similar experiences and support needs to the ethnic majority. Ethnic specific factors only emerged in the last theme when participants expressed potentially higher levels of shame and stigma. Shefer et al. [22] argued that ethnic minority groups experience more stigma and shame within their community than the ethnic majority regarding mental health issues, which in turn affects help-seeking behavior for such issues [23]. A previous study by Barnes [16] in the US revealed that African American individuals bereaved by suicide often had no support system due to the stigma associated with suicide. Participants reported that, in order to avoid stigma, they would not discuss the suicide further [16]. However, the data collected as part of the current study could not indicate whether lack of support was linked to the ethnic background of participants or was simply a reflection of the overall lack of support offered to individuals bereaved by suicide. Indeed, the current analysis did not directly compare the sample used in this study to a White British sample.

### 4.2. Limitations

This is the first study in the UK looking at the experiences and needs of minority ethnic groups in relation to suicide bereavement. This specific focus constitutes the main strength of the study. However, some of its limitations should be addressed. This study discussed the experiences of ethnic minority communities as a homogeneous group and doing so may have led to a failure to identify differences between specific groups (e.g., between Asian and Black communities). It could be that certain experiences (e.g., heightened stigma) were specific to one group, which the design of this study could not identify. In addition, there were no specific questions on the views and needs of minority groups which may have elicited further material on differences between groups. No data on immigration was collected as part of this study, although previous research has evidenced that immigrant populations may present different help-seeking patterns from the majority population for mental health issues [30]. Our data also did not allow us to draw conclusions regarding the presence of external stigma as defined by Shefer et al. [22]. Future research could thus benefit from investigating how immigration and external stigma may impact the needs of individuals bereaved by suicide in ethnic minority groups. Finally, it is important to note that snowball sampling may have limited the representativeness of the sample and that 21.6% of the sample had been bereaved for over ten years. Thus, their experiences may not reflect current support provision in the UK.

### 4.3. Future Research and Practical Implications

This study begins to explore potential differences in experiences of suicide bereavement and support needs between the ethnic majority and ethnic minority groups but it was, nonetheless, unclear if aspects of suicide bereavement were more specific to or more prevalent in ethnic minority groups, as well as differences between these groups. The experiences of these communities would benefit from further research to ensure that our understanding of their experiences and needs is comprehensive and accurate.

The findings suggest that ethnic minority groups require services that are accessible and visible, and that can address their specific needs, especially to tackle stigma. Some participants felt that professionals failed to engage with them and, therefore, that there may be a need to develop support and services that are culture-specific. It is important to note that participants did report engaging with services when given the opportunity. This is a significant finding in relation to the development of postvention services. In October 2019, the Department of Health and Social Care revealed that, as part of the NHS Long-Term Plan, the government would provide GBP 1 million to ten areas across England to develop suicide bereavement support [12]. Such support will be made available to relatives and friends of the deceased shortly after the death. It could include individual sessions with trained counsellors or volunteers, support groups, or referrals to appropriate mental health services. The support offer will differ between areas to link with already available services, such as local charities, the police, coroners, and healthcare professionals. Further research will need to clarify whether support for the ethnic majority in the UK is appropriate for people from a minority ethnic background. However, the preliminary findings of this study suggest that offering immediate accessible support, as described in the NHS Long-Term Plan, to the bereaved could be a first solid step towards better support provision for ethnic minority groups. It is hoped that similar policies will be adopted in Wales, Scotland, and Northern Ireland.

The Support After Suicide Partnership and their recently published *Core Standards for Developing and Running a Suicide Bereavement Support Service* [31] may also further support the development of postvention services for ethnic minority groups in the UK. One of these Core Standards, “Awareness and Access”, aims to ensure that services are accessible to all, and address equality and diversity issues within suicide bereavement support [31]. This study provides an insight into the needs of ethnic minority groups and, along with future research, could provide a basis for the development of evidence-based support for individuals from an ethnic minority background.

## 5. Conclusions

This is the first study in the UK to engage with ethnic minority groups and provide a preliminary understanding of their experiences and needs in relation to suicide bereavement. The data suggested that experiences reported by these communities were similar to those described in previous research on predominantly White samples. Participants reported a clear lack of support despite attempts to seek help and engage with services, and expressed the need for a coordinated and structured approach to suicide bereavement support. These initial findings suggest that providing support that is accessible and visible, tackles stigma, and enables professionals to successfully engage with these groups could make a significant difference to the experiences of people bereaved by suicide.

## Figures and Tables

**Table 1:** Free-text questions and response characteristics.

Question	Range of Response Length (in Words)	Mean Response Length (in Words)	Response Frequency *N* (%)
36. After the person had died by suicide did you feel this led to you engaging in high risk behavior? (Examples of high risk behaviors might include fighting, driving dangerously, unprotected sexual activities, excessive spending, or adopting behaviors that were similar to how the person died.) If yes, please tell us about the high risk behavior in the space provided below.	2–25	6	35 (15%)
		
		
37. If you have any other comments on how the death of this person affected you, please tell us in the space provided below.	2–642	52	103 (45%)
		
52. Are there any other organizations that you feel could have offered you help? If so, please tell us in the space provided below.	1–92	18	53 (23%)
		
		
		
53. From your experience, how and when would you like to be approached when being offered support by organizations?	1–122	14	73 (32%)
		
58. Please tell us about your experience of using support services in the space provided below.	1–145	33	68 (30%)
60. Please provide further information on why you did not access support services.	1–114	28	37 (16%)
64. Please tell us anything else you think might help to improve support for people bereaved or affected by suicide.	1–353	41	55 (24%)

**Table 2:** Ethnic background of participants.

Ethnic Group	*N*	Frequency
Mixed/Multiple ethnic groups	106	46.7%
White and Black Caribbean	36	15.9%
White and Black African	8	3.5%
White and Asian	39	17.2%
Other Mixed/Multiple ethnic background	23	10.1%
Asian/Asian British	73	32.2%
Indian	48	21.1%
Pakistani	9	4%
Bangladeshi	2	0.9%
Chinese	4	1.8%
Other Asian background	10	4.4%
Black/African/Caribbean/Black British	32	14.1%
African	9	4%
Caribbean	19	8.4%
Other Black/African/Caribbean background	4	1.8%
Other ethnic group	16	7%
Arab	2	0.9%
Any other ethnic background	14	6.2%

## Data Availability

Research data are not shared.
